# Individualism and social solidarity in vaccination policy: some further considerations

**DOI:** 10.1186/s13584-017-0147-2

**Published:** 2017-04-06

**Authors:** Fiona M. Sim

**Affiliations:** Royal Society for Public Health, University of Bedfordshire; London School of Hygiene & Troical Medicine, London, UK

## Abstract

This commentary, in response to the paper by Boas et al [IJPHR December 2016], considers some of the wider ethical, cultural and practical factors that may influence the official response of a polio-free nation following the identification of introduced wild virus within its borders. It looks at factors influencing vaccine uptake internationally, using examples of nations striving to improve childhood vaccine uptake, the relevance of mandatory versus voluntary immunisation and the role of public education and misinformation.

## Background

The paper by Boas and colleagues [1] provides an interestingly broad perspective on an important public health issue, that of vaccine uptake, and value was added by the breadth of disciplines among the authors. The description of the challenges encountered in striving to achieve a reasonable level of vaccine uptake in a situation of increasing risk were well set out and this reader found the analogy with the military a particularly fascinating and thought-provoking strand.

## Context

Global efforts to eradicate polio continue. It is a highly infectious disease that mainly affects youngchildren and can cause severe paralysis [2] with long term disability. With no effective treatment, prevention through safe mass immunisation is for most of us, including WHO, the obvious rational strategy towards control and eradication. Despite decades of coordinated and sustained global effort, polio remains endemic in three countries, Afghanistan, Pakistan and Nigeria [3] . The WHO has recognised that lack of security is a risk factor for continuing wild virus transmission [3 ibid]. This brings about the common association with poor access to immunisation as well as a shortage of other essential services, which may become dispensable in times of conflict. The WHO European Region was declared polio-free in 2002, but the ongoing presence of under-vaccinated groups and communities means that the possibility of an outbreak of clinical infection remains.

Effective surveillance is key to the eradication campaign. Israel’s surveillance system, which led to the recognition of circulating wild virus in 2013 and thence the response described in Boas and colleagues’ paper, has been recognised by WHO as “a well-functioning environmental surveillance system” [4].

## Managing the risk: improving vaccine uptake

It was the response to surveillance-detected transmission of wild virus that led to the urgent immunisation campaign described in Boas et al’s paper. The campaign they describe was based throughout upon voluntary compliance with immunisation. Here, the authors might have described how alternative strategies adopted elsewhere in the world at various times may have been considered and ruled out. For example, immunisation has been made mandatory at times in various situations in different jurisdictions. Examples include the mandatory immunisation of health workers, largely to protect the people they serve and themselves - against hepatitis B for example; in some places seasonal influenza immunisation has been made compulsory for health care workers, whilst in others the uptake of this vaccine has been encouraged or incentivised by employing authorities with varying degrees of success. For childhood immunisation, children in the USA may not commence elementary school without evidence of completion of the primary immunisation schedule [5], although there have to be exclusions for the small number of children for whom immunisation may be genuinely clinically contraindicated. In some States, exemptions may also be granted for religious and other reasons, which has tended to result in clustering of unimmunised groups and greater risk in those areas of vaccine preventable disease. So when we refer to mandatory immunisation, the term is open to different definitions and a variety of exemptions, and is often a strong recommendation rather than a requirement actually enforced in law.

The first VENICE study of immunisation programmes across Europe conducted in 2007 found that “Of the 28 participating countries, ten reported mandatory vaccinations for different vaccines in their national immunisation programmes.” [6]. By the second study in 2010, the findings among the 29 participating countries [all 27 EU countries and Norway and Iceland] were that “in total 15 countries do not have any mandatory vaccinations; the remaining 14 countries have at least one mandatory vaccination included in their programme. Vaccination against polio is mandatory for all children in 12 countries” [7]. The authors noted the need to understand different definitions of ‘mandatory’ across nations, but noted that, in some circumstances, immunisation could be imposed by legislation. Within countries, there may also be differences in policy - in the Veneto Region of Italy, for example, nationally mandatory childhood immunisation, including polio, became ‘recommended’ in 2007, with the policy continuously under review. Local reports of an enhanced response to immunisation following the change were interesting [8]. Overall, the VENICE authors noted that the reasons for different policies tend to be historical and cultural, rather than evidence-based. So the Veneto example may provide some welcome evidence going forward. However, Italy published a revised national immunisation plan in 2015, which is likely to further complicate any analysis.

Whether or not recipients - or their parents - have to pay for immunisation is another factor that may influence uptake. Cost is likely to be a serious deterrent in the least affluent communities and a major contributor to persistent inequity. In Israel, there is no direct financial cost to families to have their children immunised. However, time is a cost and therefore a potential deterrent to participation, particularly for working parents.

An important element of Israel’s response as described by Boas et al was the decision to promote immunisation as being of benefit to the family. The ethical considerations here are of interest. Boas and colleagues’ paper describe the ethical issue in the Israeli response as solidarity, with “the family as a metaphor for social solidarity”. The analogy with army service was fascinating, perhaps especially so from the perspective of a reader in a country where national service was abolished well over 50 years ago [the last UK conscripts joining up in 1960]. This analogy may well have been highly meaningful in Israel, where all young men and women are required to do national service, an obligation promoted as being for the benefit of the country as a whole. But such an analogy may be hard to deploy effectively in lands where the majority of today’s parents have no recall of conscription. Even in Israel, the military comparison might be a less than helpful one, since the risk of injury of death during national service in Israel, is surely considerably greater, even if low, than any risk reasonably attributable to childhood polio immunisation, which is minimal, almost zero. Thus parents may be under a misconception of inflated risk, being persuaded inadvertentlythat immunisation, like army service, does carry tangible risk to the children being immunised. Whether or not such an analogy is either reasonable or effective may deserve further research.

Boas et al. illustrate very convincingly the role of individual health professionals in gaining the trust of the communities they serve, which was considered to be more effective than an expectation of trust in the State. This was particularly so among marginalised communities such as the desert-living Bedouin amongst whom the wild virus was initially found. It could be that such tactics would be worth emulating among similarly marginalised communities in other countries, where vaccine uptake is poor compared with the mainstream. Somewhat ironically, in the UK some of these are religious Jewish communities, where trust in the State is low and accompanied by lack of appreciation of the protection afforded to the whole community - and indeed to families - through mass vaccination. Campaigns to increase vaccine uptake among a north London Charedi community have recently become focussed with a tailored campaign to reach out to those communities [9].

## More on ethical principles

Whilst Boas and colleagues have focussed legitimately on the concept of solidarity, it seems worth looking briefly at how the four conventional bioethical principles (non-maleficence, beneficence, justice and autonomy) might fit into the story. The first ethical principle taught in medical school is usually described is non-maleficence or “Do no harm”. The centrality of this principle is clear and worth holding at the centre of any individual’s clinical practice as well as being central to public health policy. Arguably, for example, this was the principle most massively breached by Wakefield and colleagues, in reporting in 1998 what have since been debunked as fraudulent research findings making an association between MMR immunisation and autism [10]. The resulting substantial fall in immunisation levels and reappearance of outbreaks of measles (including some very serious cases, particularly in the UK and other Western countries), bears witness to the influence of medical men, even when that influence is entirely misplaced. Even today, Wakefield’s influence continues, with release of a film [*Vaxxed: from cover-up to catastrophy,* 2016] made in the USA (and withdrawn from showings in the US and UK following objections led by the scientific community, though still on release), that continues to espouse the now long-discredited link between autism and MMR immunisation.

The principle of beneficence anticipates the physician making a beneficial contribution to the health of those whom s/he serves - and so the provision of immunisation to a population may be regarded as a beneficent act. The act does not have to be a gift, and the fact that, whilst some of those carrying out immunisation may be volunteers, healthcare workers are routinely remunerated by the State (or in some countries by international aid agencies) for providing such services, does not detract from the act of beneficence.

Where does justice fit? The provision of immunisation ticks the box for justice - a universal intervention provided regardless of ability to pay or rank in society. In the case of polio immunisation, additional efforts required to engage communities with poor vaccine uptake may have contributed towards some reduction in historical inequity. However, there is always the potential for tension between social justice and individual autonomy [11].

Autonomy, the fourth of the conventional bioethical principles, was well described in Boas et al’s paper by the nurse’s heartwarming description of her relationship with her Bedouin patients: having a relationship based on mutual trust, she was able to scotch the fear articulated by the community that their children were being singled out for adverse treatment in comparison with Jewish children, enabling them to own the decision to have their children immunised.

## Conclusion

Boas and colleagues have described Israel’s custom-made response to an urgent need to protect its population from polio, which resulted in 75% vaccine uptake in the target group and no clinical cases of the disease. This commentary has explored a few additional issues that may affect vaccine uptake. There is scope for further research to understand better the cultural and social factors influencing uptake of immunisation and other public health preventive interventions.
